# A Network-Based Methodology to Identify Subnetwork Markers for Diagnosis and Prognosis of Colorectal Cancer

**DOI:** 10.3389/fgene.2021.721949

**Published:** 2021-11-01

**Authors:** Olfat Al-Harazi, Ibrahim H. Kaya, Achraf El Allali, Dilek Colak

**Affiliations:** ^1^ Biostatistics, Epidemiology and Scientific Computing Department, King Faisal Specialist Hospital and Research Centre, Riyadh, Saudi Arabia; ^2^ College of Medicine, Alfaisal University, Riyadh, Saudi Arabia; ^3^ African Genome Center, Mohammed VI Polytechnic University, Benguerir, Morocco

**Keywords:** colorectal cancer, network, biomarker, omics, subnetwork, prognostic

## Abstract

The development of reliable methods for identification of robust biomarkers for complex diseases is critical for disease diagnosis and prognosis efforts. Integrating multi-omics data with protein-protein interaction (PPI) networks to investigate diseases may help better understand disease characteristics at the molecular level. In this study, we developed and tested a novel network-based method to detect subnetwork markers for patients with colorectal cancer (CRC). We performed an integrated omics analysis using whole-genome gene expression profiling and copy number alterations (CNAs) datasets followed by building a gene interaction network for the significantly altered genes. We then clustered the constructed gene network into subnetworks and assigned a score for each significant subnetwork. We developed a support vector machine (SVM) classifier using these scores as feature values and tested the methodology in independent CRC transcriptomic datasets. The network analysis resulted in 15 subnetwork markers that revealed several hub genes that may play a significant role in colorectal cancer, including *PTP4A3*, *FGFR2*, *PTX3*, *AURKA*, *FEN1, INHBA*, and *YES1*. The 15-subnetwork classifier displayed over 98 percent accuracy in detecting patients with CRC. In comparison to individual gene biomarkers, subnetwork markers based on integrated multi-omics and network analyses may lead to better disease classification, diagnosis, and prognosis.

## Introduction

Artificial intelligence (AI) and Machine learning (ML) approaches have been widely used to investigate the disease diagnosis and predict the outcome (Maciukiewicz et al., 2018; Lai et al., 2019; Eicher et al., 2020; Jamal et al., 2020; Sanchez and Mackenzie, 2020; Sinkala et al., 2020; Stafford et al., 2020; Toraih et al., 2020). The integration of multiple high-throughput omics datasets, such as messenger RNA (mRNA) expression profiles, proteomics, copy number alterations (CNAs), methylation and others, may increase the robustness and reliability in identifying disease associated biomarkers (Colak et al., 2010; Colak et al., 2013; List et al., 2014; Al-Harazi et al., 2016; Colak et al., 2016; Aldosary et al., 2020; Eicher et al., 2020). A protein−protein interaction (PPI) network can be defined as a directed or undirected network that consists of vertices as proteins or genes and edges as the interactions among them (Wiredja and Bebek, 2017; Sanchez and Mackenzie, 2020). Interactions among proteins or genes are meant to be specific and biologically meaningful (Wiredja and Bebek, 2017; Sanchez and Mackenzie, 2020). It has been reported that network-based approaches have high efficacy in identifying biomarkers for numerous complex diseases, including cancers (Wang et al., 2017; Chen et al., 2019; Liu et al., 2019; Uddin et al., 2019; Khan et al., 2020; Van et al., 2020).

Traditional statistical approaches are not suitable for detecting gene interactions, especially when interactions appear between more than two genes, or when the data are high-dimensional, meaning the data have many attributes or independent variables (McKinney et al., 2006; Lai et al., 2019). Machine learning approaches have been widely used to identify disease biomarkers (Lim et al., 2019; Moni et al., 2019; Tabl et al., 2019; Sanchez and Mackenzie, 2020). Recently, Sanchez *et al.* identified methylation biomarkers for leukemia by investigating PPI for differentially methylated genes (DMGs) and differentially expressed genes (DEGs) using machine learning approach (Sanchez and Mackenzie, 2020). The authors reported that the identified biomarkers are reliable and associated with cancer development and risk (Sanchez and Mackenzie, 2020). Tabl *et al.* proposed a hierarchical machine learning system to develop biomarkers that can support the identification of the best therapy for breast cancer patients based on their gene expression and clinical data that achieved a high classification accuracy (Tabl et al., 2019). Furthermore, Sinkala *et al.* applied machine learning algorithms coupled with integrative profiling of multiple data types to identify biomarkers that can differentiate between pancreatic cancer subtypes (Sinkala et al., 2020).

When a specific gene/protein is related to a particular disease or biochemical process, its associated genes/proteins may also be involved in the same disease or biochemical process (Barabási et al., 2011). Most interaction networks can be clustered into small connected subgraphs that are called disease modules or subnetworks (Barabási et al., 2011). A disease subnetwork or module consists of linked genes or proteins that share mutations, biological processes or expression variations which can be related to a specific disease (Al-Harazi et al., 2016). Previous reports indicated that the development of disease-related subnetwork markers is a robust approach that can provide markers with higher accuracy in disease classification in comparison to using individual genes (Al-Harazi et al., 2016; Khunlertgit and Yoon, 2016; Al-Harazi et al., 2019). Indeed, network-based analysis of gene expression profiling was performed to identify subnetworks and hub genes that are associated with different cancer, including breast cancer (Khan et al., 2020), lung cancer (Huang et al., 2015), ovarian cancer (Zhang et al., 2013), and others and have demonstrated the significance of the method in discovering genes related to development and progression of cancer (28).

Colorectal cancer (CRC) is one of the most frequent cancers, with a high morbidity and mortality rate. In 2018, approximately 1.8 million new instances of CRC were diagnosed, and 881,000 deaths (Bray et al., 2018). Despite advances in screening and treatment strategies, the annual incidence and mortality rates of CRC are still increasing rapidly. Molecular studies have reported that CRC is a complex and molecularly heterogeneous disease (Hahn et al., 2016; Molinari et al., 2018; Murphy et al., 2019; Fanelli et al., 2020). Gene-expression profiling is widely used in developing prognostic and diagnostic signatures for colorectal cancer (Chen et al., 2016; Xu et al., 2017; Uddin et al., 2019; Zuo et al., 2019). However, because of the heterogeneity of CRC, minimum overlapping was observed in gene lists reported in previous studies (Cao et al., 2017).

In this study, we developed an integrated omics and network-based methodology to identify subnetwork markers for disease diagnosis and prognosis. We applied our methodology to develop subnetwork markers for CRC. We first performed integrated analysis of global gene expression and copy number data. We then constructed a PPI network for the identified DEGs using molecular interaction data from several databases, including Database of Interacting Proteins (DIP) (Salwinski et al., 2004), BioGRID (Chatr-Aryamontri et al., 2017), HPRD (Mishra et al., 2006), IntAct (Kerrien et al., 2007), BIND (Alfarano et al., 2005), and Molecular INTeraction database (MINT) (Licata et al., 2012). We calculated an activity score for each subnetwork and built a classifier using these scores as feature values. We finally validated diagnostic and prognostic potential of the identified network markers.

## Materials and Methods

### Data Collection and Integrated Analysis

Whole-genome gene expression and CNA datasets for Saudi patients with colorectal cancer were gathered from the NCBI GEO (www.ncbi.nlm.nih.gov/geo). The whole-genome gene expression dataset (GSE23878) contains 35 colorectal cancer and 24 noncancerous matched samples (Uddin et al., 2011). All samples were probed using Affymetrix Human Genome U133 Plus 2.0 Array. The raw data were normalized using GC Robust Multi-array Average (GC-RMA) algorithm (Wu and Irizarry, 2004; Wu and Irizarry, 2005). The differentially expressed genes (DEGs) were identified using Analysis of Variance (ANOVA) with the adjustment of probability (p) values for multiple comparisons by false discovery rate (FDR) according to Benjamini-Hochberg step-up procedure (Benjamini and Hochberg, 1995). The DEGs were defined as those with absolute fold change (FC) > 2 and adjusted *p*-value < 5%.

The CNA dataset contains thirty samples (15 tumor and 15 adjacent normal samples) from Saudi patients (GSE47204) (Eldai et al., 2013). The data were generated using Affymetrix CytoScan HD arrays. The CNAs were identified as previously described in (Eldai et al., 2013) that revealed 144 genes with copy number changes (91 of which associated with CRC, that we included in our analysis). Next, we used the Venn diagram approach to identify the DEGs with copy number alterations in CRC using data from global mRNA and CNA. These genes were then used as input list or “seed genes” for building the PPI network (Martin et al., 2010).

**FIGURE 1:**
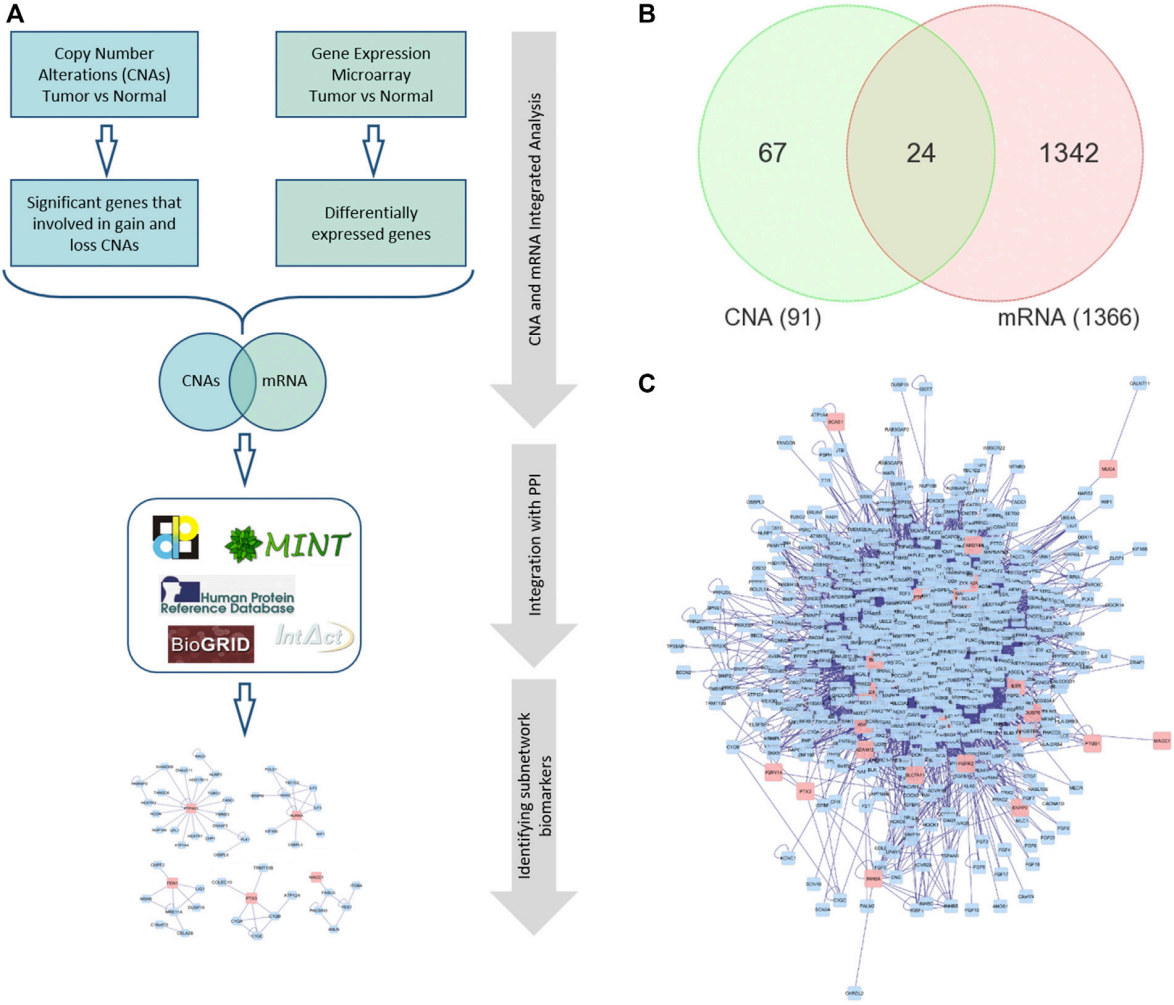
**(A)** The framework of performing integrated omics and network-based analysis. **(B)** Venn diagram representing the overlapping DEGs between global mRNA and CNA. **(C)** A PPI network for the seed/differentially expressed genes. Red nodes indicate the seed genes and the blue ones are the interacting genes. Edges represent the interactions. PPI: Protein-protein interaction.

Functional annotation and biological term enrichment analysis were performed using the Database for Annotation, Visualization and Integrated Discovery (DAVID) (Huang et al., 2009) and Protein Analysis Through Evolutionary Relationships (PANTHER) (Mi et al., 2019). Figure 1A illustrates the framework for the integrated analysis. Statistical analyses were performed using PARTEK Genomics Suite (Partek Inc., St. Lois, MO, United States). All statistical tests were two-sided and *p*-value < 0.05 was considered statistically significant.

### Protein−Protein Interaction Network Construction and Subnetwork Identification

We built the PPI network using BisoGenet, a Cytoscape plugin (Martin et al., 2010). BisoGenet imports the interaction data from several databases, including DIP (Salwinski et al., 2004), BioGRID (Chatr-Aryamontri et al., 2017), HPRD (Mishra et al., 2006), IntAct (Kerrien et al., 2007), BIND (Alfarano et al., 2005), and MINT (Licata et al., 2012). We input the set of seed genes (the DEGs with altered CNs) into the plugin which then builds the gene networks. The input list of genes (seed genes) are mapped to nodes, that will become initial set of network nodes (seed nodes) from which the network is expanded (Martin et al., 2010). The edges of the PPI network represent molecular interactions.

The constructed gene network is then clustered into subnetworks using another Cytoscape pluginclusterMaker” to cluster the network into subnetworks (Morris et al., 2011). The plugin provided the Markov Cluster Algorithm (MCL) (Van Dongen, 2001; Enright et al., 2002) that we used in our analysis. The MCL is a widely used method for analyzing complex biological networks. It uses a flow simulation to perform clustering of graphs by first building a matrix of values to be clustered that are stored in edge attributes. Then MCL algorithm is performed iteratively. After constructing the discriminant subnetworks, we selected only the subnetworks that contained at least one gene from the seed genes and the number of nodes ≥3. We then performed functional annotation and biological term enrichment analyses of the identified subnetworks using DAVID (Huang et al., 2009) and PANTHER (Mi et al., 2019).

### Scoring Subnetworks and Classification

We standardized the expression data for each gene across all samples using the z-transformation before calculating the subnetwork activity scores. We then calculated an activity score for each subnetwork as the average expression of up-regulated genes minus that of down-regulated genes in each sample. These scores were then used as feature values to build a classification model using GSE23878 dataset for training (*n* = 59). In order to assess the classifier’s performance, we used an independent microarray gene expression dataset for human colorectal cancer from The Cancer Genome Atlas (TCGA) database (TCGA data version 2016_01_28 for colorectal adenocarcinoma (COADREAD); https://gdac.broadinstitute.org/). The dataset contains 244 samples (222 tumor and 22 normal samples) performed on Agilent 244K Custom Gene Expression G4502A-07–3 arrays. We used level 3 preprocessed and normalized gene expression data as described in detail by the TCGA workgroup at the Broad Institute at the link above.

We tested the designed classifier based on the identified subnetwork markers by measuring its ability to differentiate patients from normal controls. The following measures were used for evaluating the performance:

$$\begin{array}{l} \text{Accuracy}=\frac{\text{true}\;\text{positive}+\text{true}\;\text{negative}}{\text{true}\;\text{positive}+\text{false}\;\text{negative}+\text{false}\;\text{positive}+\text{true}\;\text{negative}}\\ \text{Sensitivity}=\frac{\text{true}\;\text{positive}}{\text{true}\;\text{positive}+\text{false}\;\text{negative}}\\ \text{Specificity}=\frac{\text{true}\;\text{negative}}{\text{false}\;\text{positive}+\text{true}\;\text{negative}} \end{array}$$

Moreover, the area under the curve (AUC) with the 95% confidence interval (CI) and unsupervised principal component analyses (PCAs) are performed to further test the performance of subnetworks.

Furthermore, we compared the classification performance of the subnetwork markers with those of the previously reported CRC gene signatures as well as the DEGs identified in this study. Hence, we built several classifiers using four well-known CRC gene signatures and tested them on the same training and validation datasets. The ColoGuideEx is a gene expression classifier consisting of 13 genes designed for CRC patients at stage II (Ågesen et al., 2012). The second gene signature (ColoPrint) is 18-gene signature that is identified using whole-genome expression data and has been shown to predict high risk of recurrence in CRC patients with stage II or III (Tan and Tan, 2011). GeneFx is a 634 probe-set signature is a prognostic assay developed for patients with stage II colon cancer (Kennedy et al., 2011). The Oncotype DX, contains 12-gene signature, is also used for Stage-II CRC patients for recurrence risk prediction and guide therapy options after surgery (Clark-Langone et al., 2010).

### Survival Analysis

We performed univariate and multivariate survival analyses using the Cox proportional hazard regression model on TCGA (COADREAD) to evaluate the prognostic value of the identified subnetwork markers and their relationships with overall survival of CRC patients. The multivariate Cox regression analysis was performed to examine whether the predictive ability of the subnetwork markers was independent of other clinical factors, including age, gender, pathologic stage, and lymphatic invasion.

The prognostic risk score for each patient is calculated as the sum of the product of subnetwork score with the corresponding regression coefficient in the multivariate Cox proportional hazard regression model analysis as follows:

$$\text{Risk}\;\text{score}=\text{SS}1*\beta_{\text{Subnetwork}1}\;+\text{SS}2*\;\beta_{\text{Subnetwork}2}+\ldots +\text{SSn}*\beta_{\text{Subnetwork}\_n}$$

where SSi and $\beta_{\text{Subnetwork}\_i}$ indicate the *i*th subnetwork score and the corresponding regression coefficient in the multivariate Cox proportional hazard analysis, respectively.

After calculating the risk scores, the median risk score is used to divide patients into high and low risk groups and Kaplan-Meier method is used to plot the survival curves. Significance between survival curves was calculated by the log-rank test. A *p*-value < 0.05 was considered statistically significant.

For further validation, we used an independent microarray dataset (GSE17537, *n* = 55) that included data from 55 CRC patients, downloaded from the NCBI GEO database and standardized using z-score transformation. We performed survival analysis using the same regression coefficients (β_i_s) that was calculated using the TCGA cohort.

## Results

### Identification of Overlapping Colorectal Cancer Differentially Expressed Genes

We first analyzed global mRNA expression profile from CRC (*n* = 35) and normal samples (*n* = 24) and identified 1,366 DEGs (up- or down-regulated) in tumor compared to normal (adjusted *p* value < 5% and absolute fold change >2). We obtained 91 significant genes identified in CNA regions from (Eldai et al., 2013) and performed Venn diagram approach to identify overlapping significant mRNAs that have concomitant copy number alterations (Figure 1B). The integrated omics analysis revealed 24 significant DEGs. Functional analysis using PANTHER (Mi et al., 2013) revealed that these genes are related to protein phosphorylation, locomotion, system process, cell migration, and cell motility, that are known to be associated with cancer (Yamaguchi et al., 2005; Singh et al., 2017; Stuelten et al., 2018) (Supplementary Table S1).

### Disease-Associated Subnetwork Markers

**FIGURE 2:**
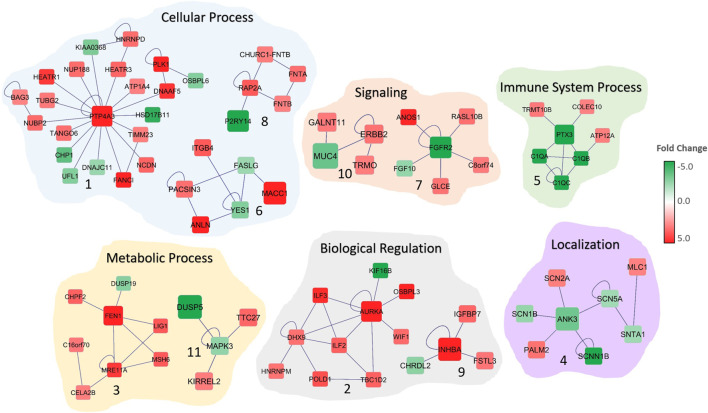
Significant subnetworks associated with colorectal cancer. The color of each gene scales with the fold-change in gene expression in CRC patients compared to normal. Up-regulated genes are indicated in red and down-regulated genes in green. Edges represent the interactions. Biological processes that are associated with the subnetworks are shown.

Several reports have demonstrated that subnetwork markers are more reliable and robust than single biomarker genes and achieved higher accuracy in disease classification (Al-Harazi et al., 2016; Al-Harazi et al., 2019). Here, we constructed gene interaction network using BisoGenet (Martin et al., 2010) for the DEGs with altered CN (seed genes; shared genes in the Venn diagram in Figure 1B). The PPI network for the seed genes had 797 nodes and 9,634 edges (Figure 1C). The PPI networks are necessary to almost all cell processes, therefore investigating PPIs is essential for understanding the physiological function of human cells in normal and disease states (Al-Harazi et al., 2016). The edges of the PPI network represent molecular interactions annotated in DIP (Salwinski et al., 2004), BioGRID (Chatr-Aryamontri et al., 2017), HPRD (Mishra et al., 2006), IntAct (Kerrien et al., 2007), BIND (Alfarano et al., 2005), and MINT (Alfarano et al., 2005) databases. The constructed PPI network is then clustered using the MCL algorithm of clusterMaker app in Cytoscape that revealed 174 gene-clusters (subnetworks). We selected 15 subnetworks that contained at least one seed gene (DEG) and the number of nodes ≥3 (Figure 2 and Supplementary Table S2).

The functional and gene ontology enrichment analyses revealed that these subnetworks are highly enriched in biological processes that are related to sodium ion transport, nuclear division, signaling, mitotic cell-cycle, biological regulation and cell communication (Figure 2; Table 1). The enriched cellular components include extracellular matrix, anchoring junction, and cytoplasm. Protein binding, growth factor binding, sodium channel activity, and voltage-gated sodium channel activity are the significantly enriched molecular functions among the 15 subnetwork markers (Table 1).

**TABLE 1:** Gene ontology enrichment analysis of 15 subnetworks.

Biological Processes	FE^a^	*p*-value	Cellular Components	FE^a^	*p*-value	Molecular Functions	FE^a^	*p*-value
Sodium ion transport	16.6	1.8E-05	Node of Ranvier	59.6	1.4E-06	Protein binding	1.3	4.9E-08
Nuclear division	4.0	4.8E-03	Extracellular matrix	4.4	2.0E-05	Growth factor binding	9.1	6.7E-05
Signaling	2.0	5.8E-03	Anchoring junction	3.3	2.0E-04	Sodium channel activity	18.5	8.8E-05
Cell cycle	3.1	8.5E-03	Cytoplasm	1.3	3.4E-04	Voltage-gated sodium channel activity	27.2	2.5E-04
Cell communication	1.9	1.0E-02	Main axon	12.3	3.9E-04	Small molecule binding	2.0	6.5E-04
Cellular response to stimulus	1.8	1.1E-02	Cell-cell contact zone	11.0	5.9E-04	Cytoskeletal protein binding	2.7	1.0E-03
Mitotic cell cycle	3.8	1.2E-02	Cell junction	2.1	1.1E-03	ATP binding	2.2	2.8E-03
Biological regulation	1.5	1.4E-02	Early endosome	3.8	2.6E-03	Kinase binding	2.7	4.2E-03
Regulation of cell communication	2.4	1.8E-02	Cell-cell junction	3.3	3.0E-03	Receptor ligand activity	3.0	1.0E-02
Cell surface receptor signaling pathway	2.3	1.9E-02	Cytoskeleton	1.9	3.7E-03	Signaling receptor activator activity	2.9	1.1E-02

a FE, Fold Enrichment is calculated by dividing the number of genes in 15 subnetworks implicated in each GO term by the expected number.

### Optimal Support Vector Machine Classification Model and Performance Comparison

We assessed the classification performance of the classifier that is designed using 15 subnetwork markers. The CRC/normal transcriptomic dataset (GSE23878) has been used as the input data for training the classifier. Expression values for each gene across all samples were normalized using z-transformation. A subnetwork activity score is then computed for each sample, as detailed in the Materials and methods section. We then designed an SVM classifier (Chang and Lin, 2011) using the 15 subnetwork scores as features to build the classification model. To evaluate the classifier’s performance, an independent microarray dataset from TCGA was used. The subnetwork markers achieved 98% accuracy, 98% sensitivity and 100% specificity, and 0.99 AUC (Table 2).

**TABLE 2:** Disease classification results of SVM classifiers using 15 subnetwork markers and other known gene signatures.

	Accuracy (%)	Sensitivity	Specificity	AUC
15 Subnetworks	98	0.98	1.00	0.99
24 DEGs*	97	0.94	1.00	0.97
ColoGuideEx	84	0.83	1.00	0.91
ColoPrint	84	0.82	1.00	0.91
Genefx	98	0.98	1.00	0.99
Oncotype DX	87	0.85	1.00	0.93

Abbreviations: AUC, Area Under Curve; DEG, Differentially expressed genes. *DEGs with copy number alterations identified in this study (Figure 1B). All classifiers for gene signatures, ColoGuideEx (59), ColoPrint (60), Genefx (61), and Oncotype DX (62) and 24 DEGs, are designed using GSE23878 dataset as training and TCGA dataset as validation.

For comparison to other gene signatures, we designed classifiers for the 24-gene DEGs (DEGs with altered CN, in Figure 1B) and four well-known gene signatures for colorectal cancer, namely ColoGuideEx (Ågesen et al., 2012), ColoPrint (Tan and Tan, 2011), Genefx (Kennedy et al., 2011) and Oncotype DX (Clark-Langone et al., 2010), using the same training (GSE23878) dataset and tested each classifier’s performance on the TCGA dataset. The results demonstrated that the subnetwork markers outperformed the 24-gene DEGs and all tested gene signatures, except for the Genefx (634–probe set signature) that achieved the same performance with our subnetwork markers (Table 2). The 15 subnetwork markers and the tested gene signatures shared only two genes: *INHBA* (Oncotype DX) and *MACC1* (Genefx).

**FIGURE 3:**
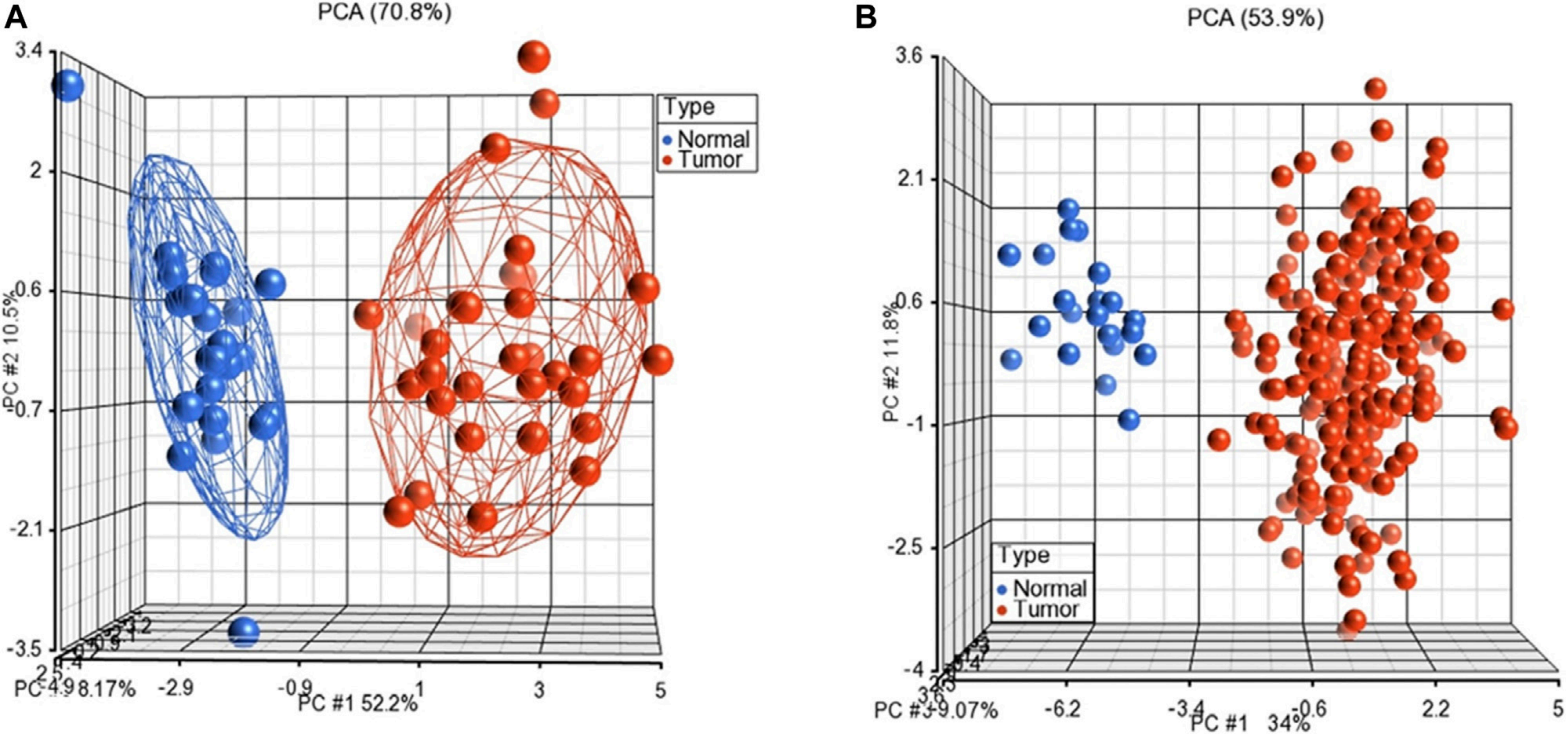
Unsupervised principal component analyses (PCAs) discriminates samples as tumors and normal controls on GSE23878 **(A)** and TCGA **(B)**. The red spheres refer to tumors and blue ones for normal controls.

Furthermore, we performed unsupervised PCA to test the performance of the subnetwork markers on GSE23878 and TCGA datasets. The PCA scatter plots, in which each sphere denotes a sample in the dataset, clearly distinguished CRC patients from normal controls in both datasets (Figure 3).

### Prognostic Risk Score and Multivariate Analysis

We performed univariate and multivariate survival analyses using the Cox proportional hazard regression model using the TCGA dataset (*n* = 222 tumor samples). We calculated the prognostic risk score for each patient as the weighted sum of subnetwork score with their corresponding regression coefficient in the multivariate Cox proportional hazard regression model analysis. The 15 subnetwork marker-risk score for each patient in TCGA data is defined as:

Risk score=SS1∗(−1.4)+SS2∗(−1.1)+SS3∗(−1.3)+SS4∗(−1.4)+SS5∗(−1.0)+SS6∗(1.2)+SS7∗(1.0)+SS8∗(−1.2)+SS9∗(1.1)+SS10∗(1.0)+SS11∗(−1.2)+SS12∗(1.0)+SS13∗(−1.2)+SS14∗(1.0)+SS15∗(−1.9)

where SSi indicates the i^th^ subnetwork score. The median risk score (−0.05) is used to divide the patients cohort into high and low risk groups.

**FIGURE 4:**
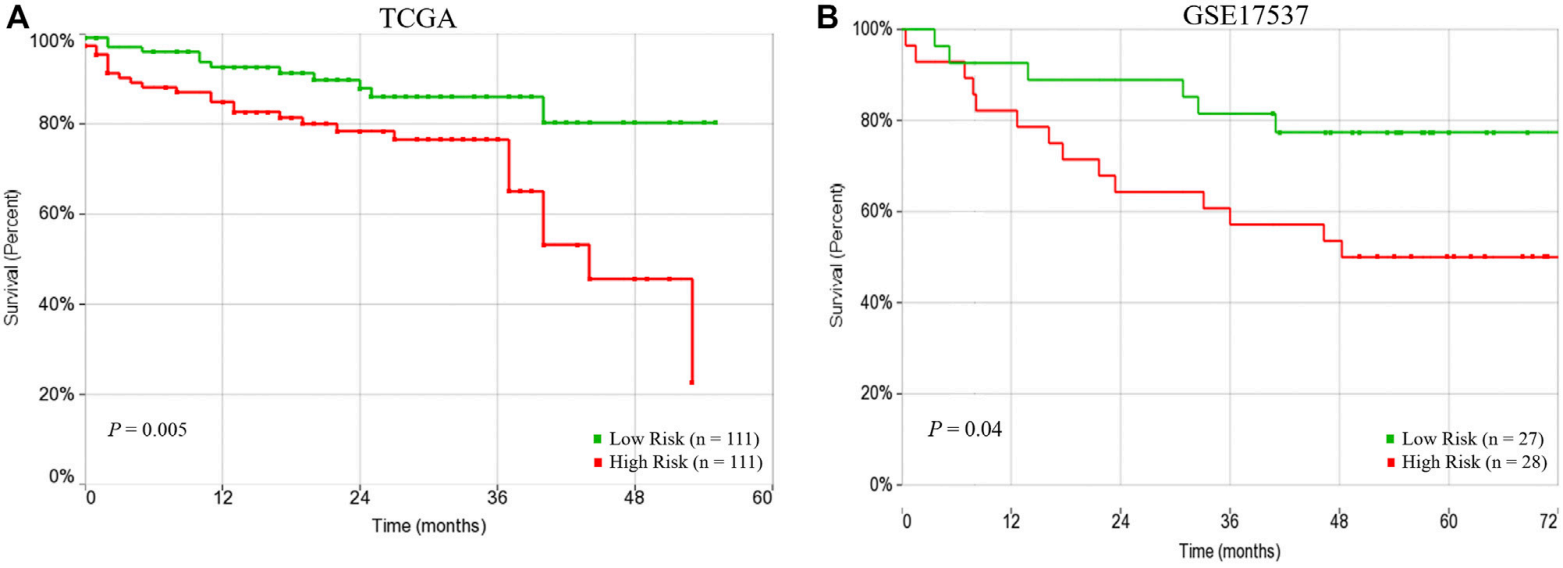
Prognostic performance of risk score based on 15-subnetwork marker in TCGA and GSE17537 CRC cohorts. Kaplan-Meier survival analysis using the 15-subnetwork markers on TCGA cohort (*n* = 222) **(A)** and GSE17537 (*n* = 55) **(B)**. Kaplan-Meier curves for risk groups, red/green curves indicate high/low-risk groups, respectively.

The univariate Cox regression analysis revealed that three factors, the 15 subnetwork markers risk score (HR = 2.53, 95% CI = 1.29–4.99, *p* = 0.007), pathologic stage (HR = 3.45, 95% CI = 1.78–6.70, *p* = 0.0003) and lymphatic invasion (HR = 2.81, 95% CI = 1.39–5.68, *p* = 0.004) were significantly associated with the CRC patients’ overall survival, but other factors did not exhibit any association with the survival (Table 3). In the multivariate analysis, the subnetwork markers showed prognostic significance for CRC overall survival risk (HR = 2.67, 95% CI = 1.35–5.30, *p* = 0.005). Hence, the multivariate Cox regression analysis revealed that the prognostic risk score based on the 15-subnetwork markers predicted the outcome of CRC independent of other clinical factors (Table 3). Indeed, Kaplan–Meier survival analysis displayed that patients in the high-risk group had a significantly poorer prognosis compared to low-risk group (*p* = 0.005) (Figure 4A). Furthermore, we used another independent dataset (GSE17537, *n* = 55) to perform overall survival analysis using the same risk score model with beta coefficients that were calculated on TCGA dataset, that also revealed significant survival differences between high and low risk groups (*p* = 0.04) (Figure 4B), confirming the prognostic significance of the 15-subetwork markers.

**TABLE 3:** Univariate and multivariate analysis associated with overall survival.

Variables	Univariate analysis		Multivariate analysis	
	*p* value	HR (95% CI)	*p* value	HR (95% CI)
**Age (years)**	0.80	0.86 (0.26–2.80)	0.86	1.12 (0.33–3.79)
≥50 vs < 50				
**Gender**	0.26	1.43 (0.76–2.66)	0.93	1.03 (0.53–2.01)
Female vs Male				
**Pathologic Stage**	**0.0003**	3.45 (1.78–6.70)	**0.01**	2.63 (1.22–5.66)
III-IV vs I-II				
**Lymphatic Invasion**	**0.004**	2.81 (1.39–5.68)	0.17	1.75 (0.78–3.90)
Yes vs No				
**Risk score**	**0.007**	2.53 (1.29–4.99)	**0.005**	2.67 (1.35–5.30)
High vs Low				

Bold indicates significance. Abbreviations: CI, confidence interval; HR, hazard ratio.

## Discussion

Integration of biological information, especially from biological networks is considered an important step for achieving more robust, stable and interpretable biomarker signature discovery (Al-Harazi et al., 2016; Alcaraz et al., 2017; Ma et al., 2019; Khan et al., 2020; List et al., 2020; Seifert et al., 2020; Sinkala et al., 2020). In this study, we proposed an integrated omics (mRNA and CNA) and network-based methodology to identify subnetwork markers. We applied our method to investigate colorectal cancer data from Saudi patients and identified 15-subnetwork markers that are associated with the disease and validated its diagnostic and prognostic potential using independent datasets.

The network-based markers have been shown to be effective in disease classification, (Zhang et al., 2013; Al-Harazi et al., 2016; Al-Harazi et al., 2019; Khan et al., 2020). Several molecular interaction databases, including DIP (Salwinski et al., 2004), BioGRID (Chatr-Aryamontri et al., 2017), HPRD (Mishra et al., 2006), IntAct (Kerrien et al., 2007), BIND (Alfarano et al., 2005), and MINT (Alfarano et al., 2005) databases have been used to construct the PPI network. Network-based methodologies are widely used for the prediction of potential candidate genes and in the construction of gene regulatory networks for different diseases (Nair et al., 2014; Dai et al., 2020; Wang et al., 2021). It has been reported that network-based methods are more effective in discovering cancer biomarkers if integrated with omics datasets (Al-Harazi et al., 2016; Cao et al., 2017; Al-Harazi et al., 2019; List et al., 2020). Indeed, our CRC associated 15-subnetwork markers that we identified in this study achieved excellent accuracy in disease classification that was better than that of several well-known colorectal cancer prognostic gene signatures, such as ColoGuideEx (Ågesen et al., 2012), ColoPrint (Tan and Tan, 2011) and Oncotype DX (Clark-Langone et al., 2010) as well as the 24-gene DEGs. In addition, our results also demonstrated the markers’ prognostic significance, hence supporting the conclusion that subnetwork markers based on integrated multi-omics and network analyses may provide robust biomarkers for disease classification, diagnosis, and prognosis. Results from the gene ontology enrichment analysis revealed enrichment of genes involved in cancer related biological processes such as protein phosphorylation (Singh et al., 2017), cell motility (Stuelten et al., 2018), and cell migration (Yamaguchi et al., 2005).

We identified subnetwork markers using the DEGs with altered CN as seed genes while building the gene network. Previous studies have indicated that integrating gene expression with CN data may lead to key cancer driver genes that are involved in tumor initiation and progression (Colak et al., 2010; Colak et al., 2013; Ohshima et al., 2017). Our integrated omics with the network-based analysis revealed potential subnetwork markers for CRC that may play an important role in tumorigenesis. The *PTP4A3* gene*,* the hub gene in Subnetwork 1 (Figure 2) was previously identified as a metastasis biomarker for the colorectal cancer (Guzińska-Ustymowicz et al., 2011). A recent study indicated that frameshift mutation in *ANK3* (hub in Subnetwork 4) in colon cancer (Yeon et al., 2018). *PTX3* (hub gene in Subnetwork 5) is involved in immune system process and has been shown to be prognostic marker for CRC (Liu et al., 2018). In another study, *FGFR2* (hub gene in Subnetwork 7) is reported to promote the PD-L1 expression via the JAK/STAT3 signaling in colorectal tumors and associated with disease progression and poor survival (Carter et al., 2017; Hu et al., 2019). Network analysis also indicated other cancer-associated genes, such as *ITGB4* and *FGF10*. *ITGB4* is considered to be a therapeutic target and prognosis marker for colon cancer (Li et al., 2019). The high expression of *FGF10* is found to be correlated with the size of the CRC tumors, indicating its critical role in the prognosis and survival of colorectal cancer patients (Farajihaye Qazvini et al., 2019). Similarly, *AURKA* (Jacobsen et al., 2018), *BAG3* (Li et al., 2018), *NUBP1* (Liu et al., 2017), and *ANLN* (Wang et al., 2016) are all found to be dysregulated in colorectal cancer and involved in cancer progression and invasion. The subnetworks revealed genes that are previously reported as CRC-associated as well as several yet undeciphered genes that may contribute colorectal cancer, such as *DNAAF5*, *RASL10B*, *DUSP19,* and *TTC27.*

In conclusion, our results demonstrated that the subnetwork markers based on integrated omics (genomics and transcriptomics datasets) are robust as disease biomarkers and may lead to better disease diagnosis and prognosis compared to single genes.

## Data Availability

Publicly available datasets were analyzed in this study. These data can be found here: The Cancer Genome Atlas (TCGA) and the NCBI Gene Expression Omnibus.
