# Supplementary material for: A Network-Based Methodology to Identify Subnetwork Markers for Diagnosis and Prognosis of Colorectal Cancer
Source: Front Genet. 2021 Nov 1;12:721949. doi: 10.3389/fgene.2021.721949 (PMC8591094; doi:10.3389/fgene.2021.721949)
Supplement: Supplementary file 2 [file Table1.DOCX]

Supplementary Table 1: PANTHER functional analysis of 24 overlapping genes

| Biological Process Term | P-value | Genes |
| --- | --- | --- |
| Protein phosphorylation | 1.24E-03 | FGFR2, DUSP5, INHBA, ENPP2, BCL2, ADRA2A, AURKA, IL6R, MYLK |
| Locomotion | 2.00E-03 | PTP4A3, ENPP2, ANK3, BCL2, ADRA2A, IL6R, MYLK, SLC7A11 |
| System process | 2.14E-03 | INHBA, PTP4A3, ANK3, BCL2, PTGS1, ADRA2A, MYLK, FEN1, MUC4 |
| Cell migration | 2.74E-03 | PTP4A3, ENPP2, BCL2, ADRA2A, IL6R, MYLK, SLC7A11 |
| Cell motility | 4.90E-03 | PTP4A3, ENPP2, BCL2, ADRA2A, IL6R, MYLK, SLC7A11 |
| Cell division | 5.27E-03 | FGFR2, ANK3, NEDD9, AURKA, MACC1 |
| Phosphorus metabolic process | 8.76E-03 | FGFR2, DUSP5, INHBA, PTP4A3, ENPP2, BCL2, ADRA2A, AURKA, IL6R, MYLK |
| Regulation of MAPK cascade | 9.38E-03 | FGFR2, DUSP5, INHBA, ADRA2A, IL6R |
| Regulation of cell migration | 9.72E-03 | ENPP2, BCL2, ADRA2A, IL6R, MYLK |
| Protein modification process | 1.12E-02 | FGFR2, DUSP5, INHBA, PTP4A3, ENPP2, BCL2, ADRA2A, AURKA, IL6R, MYLK, MUC4 |
